# A novel system to monitor mitochondrial translation in yeast

**DOI:** 10.15698/mic2018.03.621

**Published:** 2018-01-13

**Authors:** Tamara Suhm, Lukas Habernig, Magdalena Rzepka, Jayasankar Mohanakrishnan Kaimal, Claes Andréasson, Sabrina Büttner, Martin Ott

**Affiliations:** 1 Department of Biochemistry and Biophysics, Stockholm University, SE-10691 Stockholm, Sweden.; 2 Institute of Molecular Biosciences, University of Graz, A-8010 Graz, Austria.; 3 Department of Molecular Biosciences, the Wenner-Gren Institute, Stockholm University, SE-10691 Stockholm, Sweden.

**Keywords:** mitochondrial translation, flow cytometry, superfolder GFP, strain engineering

## Abstract

The mitochondrial genome is responsible for the production of a handful of polypeptides that are core subunits of the membrane-bound oxidative phosphorylation system. Until now the mechanistic studies of mitochondrial protein synthesis inside cells have been conducted with inhibition of cytoplasmic protein synthesis to reduce the background of nuclear gene expression with the undesired consequence of major disturbances of cellular signaling cascades. Here we have generated a system that allows direct monitoring of mitochondrial translation in unperturbed cells. A recoded gene for superfolder GFP was inserted into the yeast (*Saccharomyces cerevisiae*) mitochondrial genome and enabled the detection of translation through fluorescence microscopy and flow cytometry in functional mitochondria. This novel tool allows the investigation of the function and regulation of mitochondrial translation during stress signaling, aging and mitochondrial biogenesis.

## INTRODUCTION

In *Saccharomyces cerevisiae* the mitochondrial genome encodes eight proteins of which seven are membrane proteins and core subunits of the oxidative phosphorylation system (OXPHOS) 1. Respiratory activity, therefore, depends on mitochondrial translation, but the molecular mechanisms, regulation and timing of mitochondrial translation are largely unexplored 2. A major obstacle when analyzing mitochondrial translation in cells is the need to inhibit cytosolic translation in order to follow incorporation of radiolabeled amino acids into mitochondrial translation products 3. Such pharmacological inhibition impacts severely on cellular homeostasis and alters signaling cascades. For example, inhibition of cytosolic translation using cycloheximide leads to an increase in free amino acid levels, which in turn activates TOR signaling 4,5,6 and thus affects cell growth, nutrient signaling and life span 7,8,9. Additionally, cycloheximide treatment alters protein degradation 10 and acutely impacts on mitochondrial translation within very short time frames 11. To directly monitor mitochondrial protein synthesis without interfering with cellular protein homeostasis new experimental tools are needed.

Employing biolistic transformation we have integrated a gene encoding superfolder GFP into the mitochondrial genome. This reporter is compatible with mitochondrial respiratory function and enables the direct detection of mitochondrial translation *in vivo* as GFP fluorescence. This novel tool will facilitate future studies on the regulation and timing of mitochondrial translation.

## RESULTS AND DISCUSSION

### Integration of a superfolder GFP gene into the mitochondrial genome 

**Figure 1:**
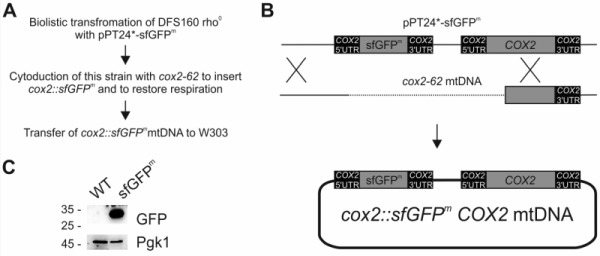
FIGURE 1: Integration of *sfGFP^m^* into the mitochondrial genome. **(A)** Strain construction strategy. **(B)** Schematic representation of integration of the reporter into the mitochondrial genome via homologous recombination. 5’ and 3’UTR of *COX2* drive the expression of sfGFP^m^. **(C)** Control of successful expression of sfGFP^m^ via Western blotting using a GFP antibody. 3-phosphoglycerate kinase (Pgk1) served as a loading control.

In a pioneering previous study, a gene encoding fluorescence-enhanced GFP was inserted into the mitochondrial genome to replace the open reading frame of *COX3* 12. The resulting strain was respiration deficient and expressed only weak fluorescence, which limited its usefulness in fluorescence microscopic and flow cytometric experiments. This limitation could be explained by poor folding of GFP expressed in the context of the mitochondrial translation system 13, which is specialized on the production of membrane proteins. To circumvent the folding problem, we employed superfolder GFP (sfGFP), which folds with enhanced kinetics resulting in a more stable protein 14. To allow translation of the mRNA by mitochondrial ribosomes its coding sequence was flanked by the authentic 5’ and 3’ untranslated regions (UTR) of *COX2* 15,16,17,18, coding for the cytochrome oxidase subunit Cox2. To avoid the respiratory deficiency associated with inactivation of mitochondrial genes, we engineered a new mitochondrial genome that coded for sfGFP as an additional ninth open reading frame. The final engineered gene was termed *sfGFP^m^* and was cloned into the pPT24* plasmid (Fig 1A-B), yielding a plasmid (pPT24*-sfGFP^m^) that contained *COX2* 5’UTR-*sfGFP^m^*-*COX2 *3’UTR as well as the authentic* COX2*, including its upstream sequences (Fig 1B). pPT24*-sfGFP^m^ was delivered into a strain that lacks mtDNA (*rho^0^*) using biolistic transformation 19. After selection, the resulting strain that carried pPT24*-sfGFP^m^ was used to cytoduce strain *cox2-62*, which lacks a functional *COX2* gene, to gain respiratory competence by homologous recombination of the two mitochondrial genomes 20. Finally, we transferred the novel mitochondrial genome into the strain W303 resulting in the strain sfGFP^m^. Western blot analysis demonstrated that this engineered genome expressed readily detectable levels of sfGFP^m^ (Fig 1C).

### Expression of mitochondrially encoded *sfGFP^m^* does not disturb mitochondrial function

**Figure 2:**
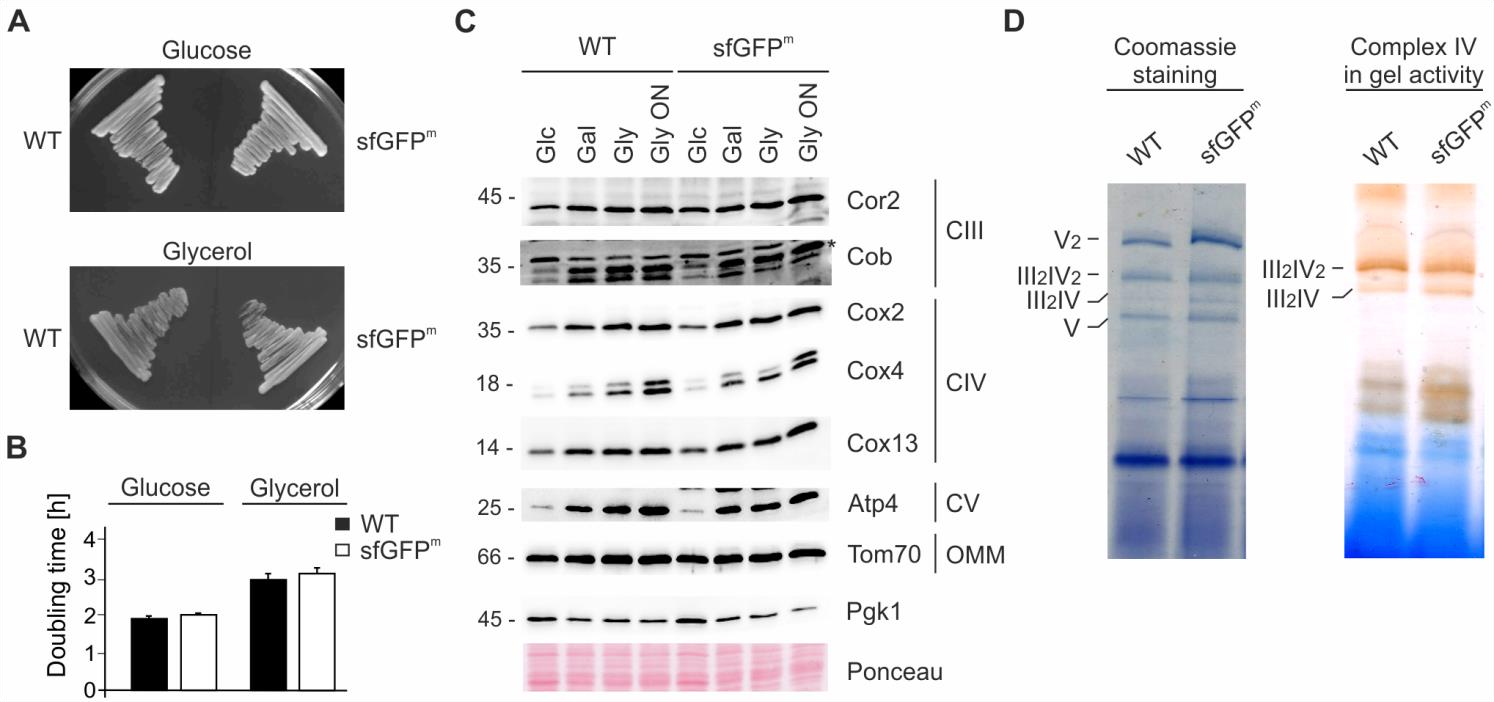
FIGURE 2: Expression of *sfGFP^m^* does not disturb mitochondrial function. **(A)** Wild type (WT) and sfGFP^m^ were streaked out on fermentable (Glucose) and non-fermentable (Glycerol) medium. **(B)** Doubling time during exponential growth phase in glucose (Glc) and glycerol (Gly). Data represent the mean of three independent experiments +/- SD. **(C)** Steady state levels of OXPHOS subunits in WT and sfGFP^m^ during exponential phase in glucose (Glc), galactose (Gal) or glycerol (Gly). Whole cell extracts were separated on SDS-PAGE and analyzed with Western Blotting using the indicated antibodies. **(D) **Isolated mitochondria of WT and sfGFP^m^ were lysed in digitonin and protein complexes were separated by blue-native PAGE. Supercomplexes (III2IV and III_2_IV_2_) were either visualized by Coomassie staining or complex IV in-gel activity assay.

We assessed the impact of *sfGFP^m^* expression on mitochondrial function by monitoring respiratory growth on non-fermentable medium and found that it was similar between wild type and the sfGFP^m^ strain (Fig 2A and 2B). As predicted, steady state levels of respiratory chain subunits were unchanged and showed the expected increase when cells were grown in non-fermentable compared to fermentable medium (Fig 2C). Finally, we checked for respiratory chain assembly into supercomplexes as well as cytochrome oxidase activity and found that both were unchanged compared to wild type (Fig 2D). We therefore conclude that expression of sfGFP^m^ does not alter the assembly of respiratory complexes or the general respiratory competence of the cells.

### Expression of sfGFP^m^ followed by flow cytometry

**Figure 3:**
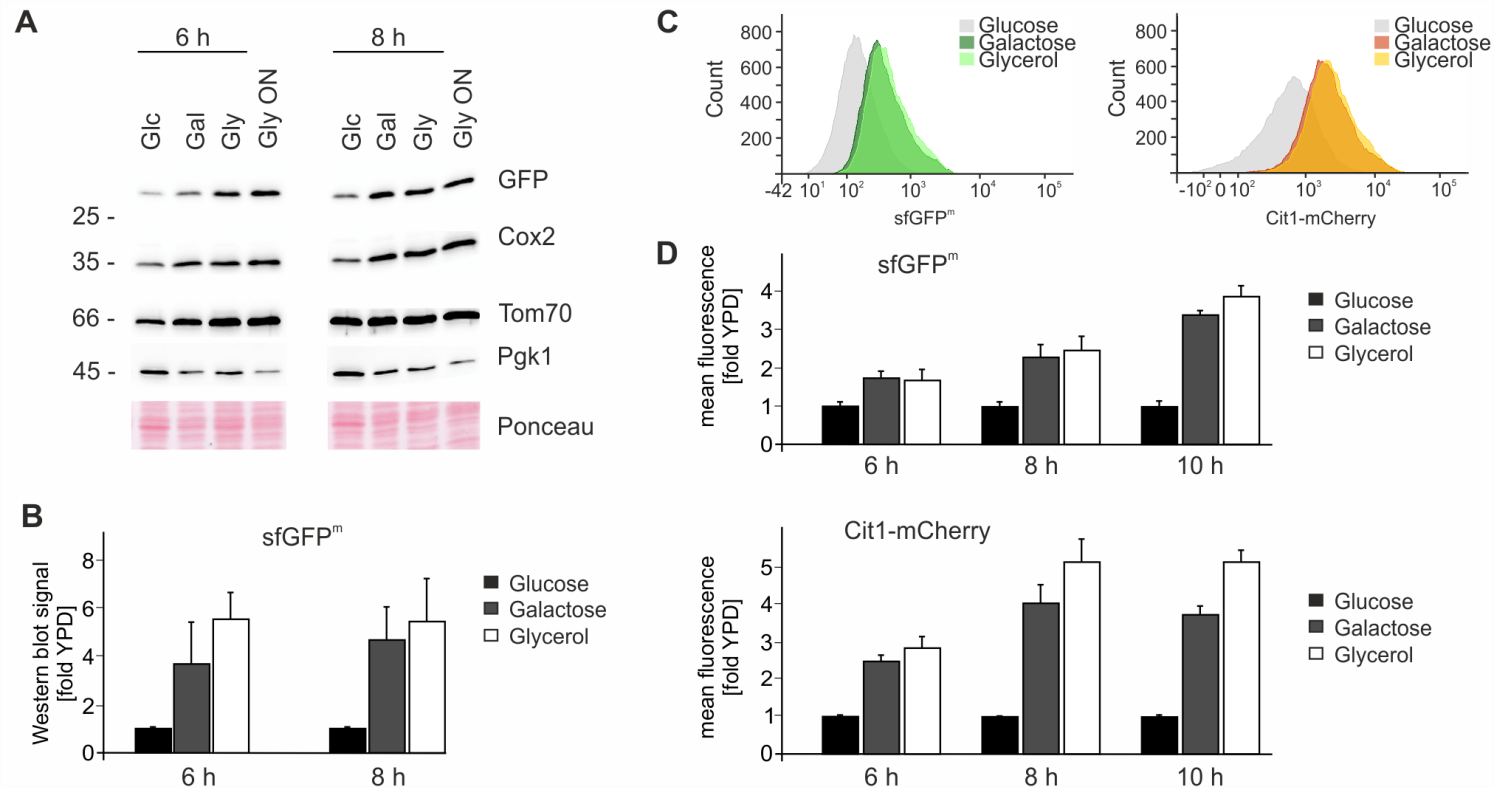
FIGURE 3: Analysis of sfGFP^m^ expression ** (A)** Cells from an overnight culture in YPD were grown to exponential phase for six or eight hours on different carbon sources. Whole cell extracts were separated on SDS-PAGE and analyzed with Western Blotting using the indicated antibodies. **(B)** Quantification of Western blotting signal of three independent experiments represented as means +/- SD. GFP signals were normalized to that of 3-phosphoglycerate kinase (Pgk1) and the YPD signal was set to 1. **(C)** Flow cytometry histogram showing sfGFP^m^ and Cit1-mCherry signals of cells grown on the indicated carbon sources. **(D)** As in A) but cells were analyzed using flow cytometry. Signal intensities of sfGFP^m^ and Cit1-mCherry were quantified from eight independent samples, and the fluorescence intensity recorded for a strain lacking any fluorescent tag was subtracted as background. Signals on galactose or glycerol were normalized to the respective time point on glucose and the mean +/- SEM is depicted.

We next explored the possibility to use sfGFP^m^ as an optical readout for mitochondrial translation. First we checked the steady-state levels of sfGFP^m^ by Western blotting during respiration and fermentation to verify that mitochondrially encoded sfGFP follows the same expression pattern as other mitochondrially encoded proteins. As expected sfGFP^m^ protein levels increased in the presence of galactose and glycerol after 6 and 8 hours in the same way as Cox2 levels (Fig 3A-B). Next we determined GFP fluorescence by flow cytometry under the same conditions and observed an increase in the fluorescent signal when cells were grown for 6, 8 and 10 hours in galactose or glycerol containing medium (Fig 3C-D). To control for mitochondrial biogenesis we used mCherry-tagged Cit1, a nuclear encoded protein that is imported into mitochondria from the cytosol 21. Similar to sfGFP^m^, Cit1-mCherry levels also increased when grown in the presence of galactose or glycerol, indicating a general increase in mitochondrial biogenesis induced by galactose and glycerol (Fig 3C-D). In sum, these results confirm that sfGFP^m^ represents a functional reporter for the analysis of mitochondrial translation *in vivo* without the need to inhibit cytosolic translation. Flow cytometry as quantitative readout opens up many possibilities to interrogate mitochondrial translation with single cell resolution and allows the combination of sfGFP^m^ with additional fluorophores for a multitude of simultaneous analyses.

### sfGFP^m^ allows detection of changes in mitochondrial gene expression

**Figure 4:**
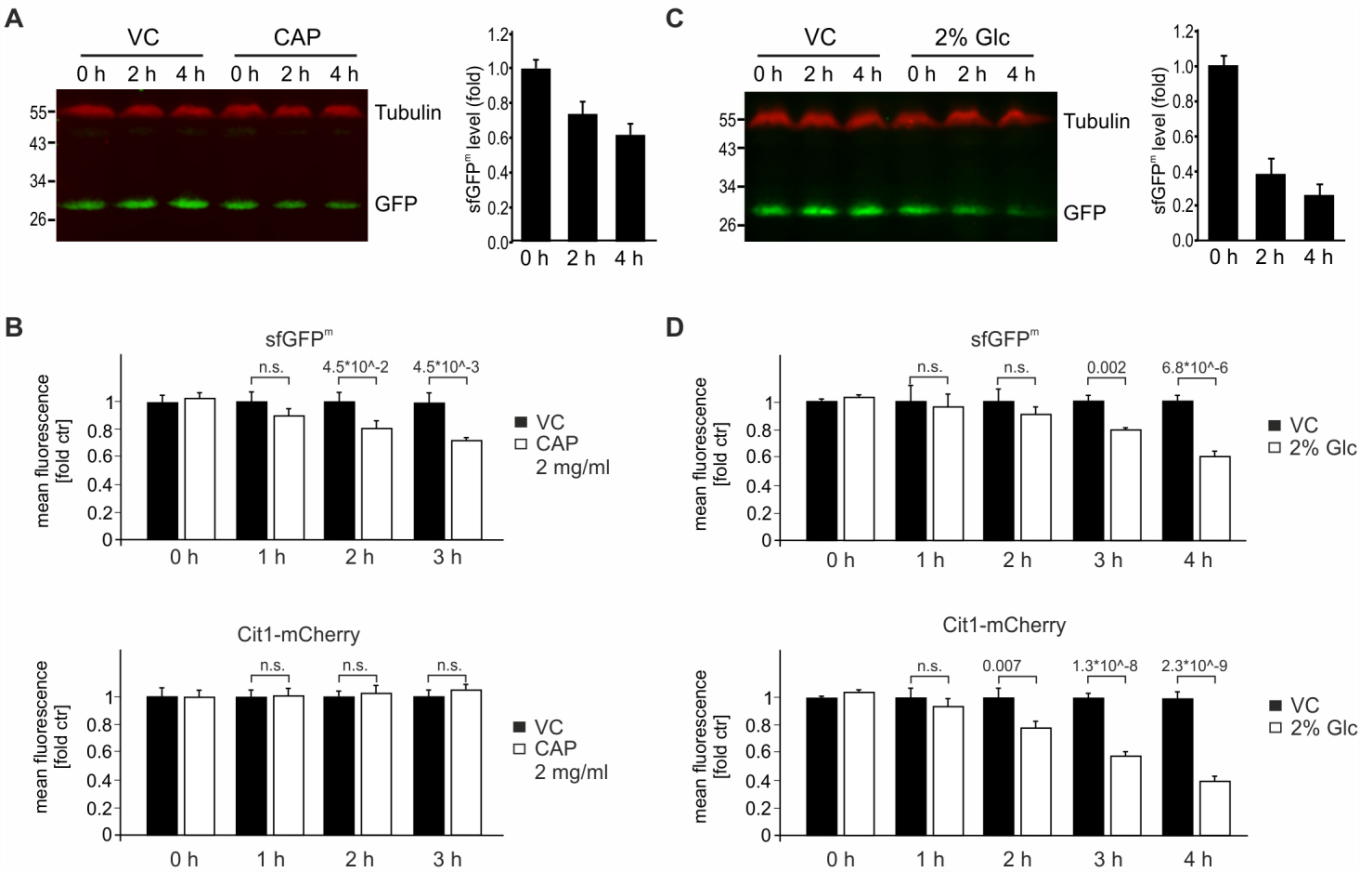
FIGURE 4: Impact on sfGFP^m^ protein levels by inhibition of mitochondrial translation or glucose repression. **(A)** Cells from an overnight culture in YPD were grown to exponential phase for six hours in glycerol. Cells were treated with 2 mg/ml chloramphenicol (CAP) or vehicle control (VC) for the indicated times and whole cell extracts were separated on SDS-PAGE and analyzed with Western Blotting using the indicated antibodies. The fluorescence signals of 6 independent experiments were quantified and the GFP signals were normalized to the tubulin signals. Data is depicted as fold of the respective untreated time point and the mean +/- SEM is displayed. **(B)** As in (A) but cells were analyzed using flow cytometry. Signals of sfGFP^m^ and Cit1-mCherry were quantified from eight independent samples, and the fluorescence intensity recorded for a strain lacking any fluorescent tag was subtracted as background before signals from treated cells were normalized to control values. Numbers indicate significance values from student t-test. **(C)** Cells were grown as in (A), but exposed to glucose. **(D) **As in (B) but cells were exposed to glucose. Numbers indicate significance values from student t-test.

We determined the stability of sfGFP^m ^by inhibiting mitochondrial translation using chloramphenicol (CAP). Using immunoblotting coupled with detection by near-infrared fluorescence, we found that sfGFP^m^ levels declined over four hours after treatment with CAP (Fig 4A). Accordingly, flow cytometry confirmed that already two hours after block of mitochondrial translation via CAP, a decrease in GFP intensity was detectable. While this loss of sfGFP^m^ signal became even more evident over time (Fig 4B), the nuclear encoded mitochondrial protein Cit1-mCherry was stable (Fig 4B). To further test the reporter construct, we made use of glucose repression of mitochondrial function 22,23,24. When adding glucose to non-fermentable medium, mitochondrial biogenesis as well as translation and OXPHOS assembly are repressed. During glucose repression, we observed a pronounced decline in sfGFP^m^ levels by Western blotting and flow cytometric evaluation. As expected, Cit1-mCherry levels were also decreased by glucose-induced repression of mitochondrial biogenesis (Fig 4C-D). Taken together, both western blot analysis and flow cytometric evaluation of sfGFP^m^ faithfully report the specific inhibition of mitochondrial translation by CAP as well as the general down regulation of mitochondrial biogenesis during glucose repression.

### sfGFP^m^ visualized by fluorescence microscopy

**Figure 5:**
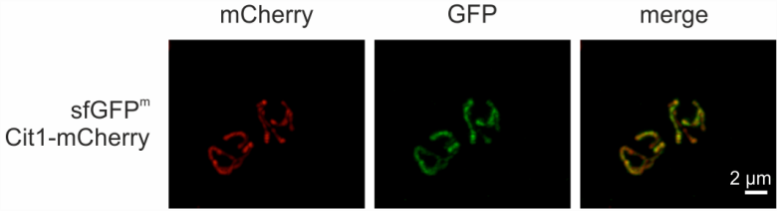
FIGURE 5: sfGFP^m^ visualized by fluorescence microscopy. Cells were grown to exponential phase in non-fermentable medium and sfGFP^m^ and Cit1-mCherry were visualized via fluorescence microscopy.

Finally, we visualized sfGFP^m^ via fluorescence microscopy. Cells grown in non-fermentable medium in exponential phase showed mitochondrially localized GFP fluorescence (Fig 5), as evidenced by co-localization with Cit1-mCherry (Supplemental movie). This opens the possibility to employ sfGFP^m^ not only as a reporter for mitochondrial translation but also to follow mitochondrial movement during cell division, mitophagy, or mitochondrial biogenesis.

In conclusion, we have established a functionally neutral mitochondrially encoded GFP reporter, termed sfGFP^m^, which is easily monitored via Western blotting, flow cytometry and microscopy. Our reporter allows studying mitochondrial translation *in vivo* without poising cytoplasmic translation and therefore enables detailed studies of mitochondrial function without perturbing cell physiology. The versatile nature of the new mitochondrial genome described here opens up new venues for the investigation of mitochondrial gene expression by optical methods in respiring cells.

## MATERIALS AND METHODS

### Generation of *sfGFP^m^*

A gene encoding superfolder GFP (sfGFP) was was recoded to match mitochondrial codon usage, synthesized (Invitrogen, Life technologies, GeneArt) and cloned into the pKM vector using NdeI and XhoI. pKM contains the 5’ and 3’UTR of *COX2* between two EcoRI restriction sites. *sfGFP^m^* flanked by the 5’ and 3’UTR of *COX2* was then re-cloned from pKM into pPT24* using the EcoRI restriction sites 15,25,26. pPT24*-sfGFP^m^ contains a part of authentic mtDNA, the sfGFP^m^ under control of the 5’ and 3’UTR of *COX2* and, downstream of *sfGFP^m^*, the full *COX2* gene including its 5’ and 3’UTR. pPT24*-sfGFP^m^ was integrated into the mitochondrial genome via biolistic transformation and homologous recombination. Briefly, the *rho^0^ kar1-1* DFS160 strain 27 was grown in YP medium containing 2% raffinose and spread on SD-Leu plates containing sorbitol (1 M). The nuclear marker plasmid pRS315 (5 µg), as well as the pPT24*-sfGFP^m^ (10-15 µg), were precipitated on tungsten particles (100 µl tungsten particle (0.5 µm), 1 M CaCl_2_, 16 mM spermidine) and spread on flying discs. The particles were bombarded onto a lawn of yeast cells with a particle gun (Biorad, USA). After four to five days positive nuclear transformants were collected on a master plate and crossed to a *cox2-62* tester strain 20,28. The diploids were screened for respiratory growth. This crossing step was repeated twice with positive clones. Eventually the stable synthetic *rho^-^* strain containing pPT24*-sfGFP^m^ was repopulated with mtDNA via cytoduction with a strain containing the *cox2-62* mutation. A haploid clone that regained respiratory competence was identified and used to transfer mitochondrial DNA into W303 *rho^0^* to create strain MOY1355 (*Mat **α **ade2-1 his3-11,15 trp1-1 leu2-3,112 ura3-1, sfGFPm::cox2, COX2*). An mCherry-tagged variant of Cit1 was generated by replacing the stop codon of the endogenous open reading frame with a sequence encoding mCherry followed by a *TRP1* selection cassette to yield strain MOY1360 (*Mat **α **ade2-1 his3-11,15 trp1-1 leu2-3,112 ura3-1,* *CIT1-mCherry::TRP1, sfGFPm::cox2, COX2*). Cells were grown at 30˚C in 1% yeast extract, 2% peptone medium supplemented with 2% dextrose (YPD), 2% galactose (YPGal) or 2% glycerol (YPG) under shaking. When indicated 2 mg/mL chloramphenicol (dissolved in ethanol) or 150 mg/mL cycloheximide (dissolved in water) were added.

### SDS-PAGE and Western Blotting

Proteins were extracted by alkaline lysis with 370 mM NaOH, precipitated with 8.33% TCA and pellets were resuspended in reducing sample buffer (50 mM Tris-HCl pH 6.8, 2% SDS, 10% glycerol, 100 mM DTT) (modified from 29). Proteins were separated using 16% acrylamide, 0.2% bisacrylamide SDS-PAGE and blotted on nitrocellulose membrane. Western blot signals were quantified using ImageJ. For immunoblot analyses upon treatment with chloramphenicol (CAP) or addition of 2% glucose, cells equivalent of 4 OD600 were harvested at indicated time points. Proteins were extracted by alkaline lysis with 1.8 M NaOH, 7.5% β-mercaptoethanol and precipitated with 27.5% TCA. Pellets were resuspended in 100 µL urea loading buffer (200 mM Tris-HCl pH 6.8, 8 M Urea, 5% SDS, 1 mM EDTA, 0.02% Bromphenolblue, 15 mM DTT) and incubated at 65°C for 10 min. Proteins were separated on 12.5% Tris-glycine SDS-PAGE and transferred onto nitrocellulose membranes. Membranes were blocked for 1 hour with 1 % skim milk in TBST (0.05% Tween) and incubated over night with antibodies directed against alpha-tubulin (ab184970) and GFP (Roche 11814460001) in TBST (0.2% Tween + 1% skim milk). Membranes were washed four times with TBST (0.05% Tween) and incubated for 1 hour with the following near-infrared fluorescent secondary antibodies: anti-mouse IRDye 800CW 926-32210 and anti-rabbit IRDye 680RD 926-68071 diluted 1:20000 in TBST (0.2% Tween + 0.01% SDS). Membranes were washed four times with TBST (0.05% Tween), rinsed twice with TBS and signals were analyzed using Odyssey Fc (Li-499 Cor Biosciences, Lincoln, NE) and Image Studio Lite software.

### Native-PAGE and complex IV activity

For native-PAGE, isolated mitochondria (100 µg) were solubilized (50 mM BisTris, 25 mM KCl, 1 mM EDTA, 2 mM aminohexanoic acid, 12% glycerol, 1 mM PMSF, 1x complete, 2% digitonin) for 10 minutes on ice. After a clarifying spin, lysates were separated on a native 3-12% Bis-Tris gel (NativePAGE, ThermoFisher). Supercomplexes were visualized by coomassie staining or complex IV activity assay. Briefly, complex IV activity was visualized by adding 2.5 mM 3,3'-Diaminobenzidine dissolved in 0.05 M phosphate buffer pH 7.4, 1 nM catalase, 1 mg/ml cytochrome *c* and 240 mM sucrose.

### Fluorescence microscopy

Cells were grown at 30°C and live images were taken using a Zeiss LSM 800 Airyscan microscope (Carl Zeiss, Jena, Germany) with a Plan-apochromatic 63X/1.4-numerical aperture oil immersion lens. For confocal excitation of GFP, a 488-nm diode laser was set at 10.0%, and emission was detected between wavelengths 492 and 540 nm. For excitation of mCherry, a 561-nm laser line was used at 2.8%, and emission was detected between 565 and 695 nm. A Z-stack of cells was performed using ZEN blue 2.1 software and maximum intensity projections of the images are shown. Image analysis was done by using ImageJ (National Institutes of Health, Bethesda, MD) and Imaris 8.4 software.

### Flow cytometry

1x10^6^ cells were harvested via centrifugation. Cells were washed once with water and resuspended in PBS (PBS, 25 mM potassium phosphate; 0.9% NaCl; adjusted to pH 7.2). For quantiﬁcation of fluorescence intensities using ﬂow cytometry (BD LSR Fortessa), 30,000 cells were recorded and analyzed with the BD FACSDiva software. A W303a strain carrying no fluorescent tag served as background, and respective fluorescence intensities on either YPD, YPGal or YPGly were subtracted as background.

### Statistics

Statistical significance was determined using student’s t-test (unpaired, two-tailed, equal variance).

## SUPPLEMENTAL MATERIAL

Click here for supplemental data file.

All supplemental data for this article are also available online at http://microbialcell.com/researcharticles/a-novel-system-to-monitor-mitochondrial-translation-in-yeast/.
